# Pills of Sulphate of Quinine

**Published:** 1853-11

**Authors:** Edward Parrish


### *On Pills of Sulphate of Quinia.*

BY EDWARD PARRISH.

Although it is not always left to the discretion of the apothecary what excipients to employ in compounding prescriptions, yet he should be so familiar with the subject as to be able to advise and instruct medical men in regard to those which are really most advantageous in the case of each of the leading remedies extemporaneously prescribed. There are few intelligent apothecaries who have not a salutary influence in modifying the views of neighboring practitioners in regard to the art of prescribing, and none who have not frequent occasion to exercise their own judgment, not only in the selection of excipients, but in other practical points in extemporaneous pharmacy.

There is, I believe, no medicine so frequently prescribed in the pilular form as the sulphate of quinia, and perhaps none, in making which into pills, there is so great a diversity of practice. The following substances are much employed as excipients for this object: Gum arabic, simple syrup, syrup of gum arabic, honey, molasses, conserve of roses, crumb of bread, flour, and simple water; and besides these, tannic acid, extract of cinchona, and various tonic, astringent and narcotic extracts, which assist, or in some way modify, the effect of the alkaloid to meet particular indications in disease.

Most pharmaceutists and medical practitioners have no doubt a preference for one or the other of them, and, as is well known, there are in standard works several formulæ indicating similar preferences.

Dr. Pereira directs the pills to be made with conserve of roses, and in the three formulæ given in the Pharmacopee Universelle, crumb of bread, honey and conserve of rose, are directed. Dorvault directs in L'Officine for di-sulphate, the extract of wormwood; for the acid sulphate, conserve of roses. The pills are not officinal in either of the British Pharmacopœias. In our own officinal directions, in the edition for 1850, gum arabic and honey are prescribed, while in that of 1840, gum arabic and syrup were the excipients.

The use of gum arabic and syrup was abandoned on account of the pills becoming insoluble, by keeping. Gum arabic and honey used together are probably less objectionable. The omission of the gum entirely is perhaps more frequently prescribed in 2, 3 and 5 grain doses than in the one grain dose, that used to be given, it is a desideratum to use an excipient which will produce the smallest possible increase of bulk at the same time that it gives a plastic mass.

The following formulæ is, I think, preferable to those in which gum arabic is employed, as well for the diminutive size as for the iucreased solubility of the pills:

Take of sulphate of quinia, 12 grains.
Powdered tragacanth, 1 grain.

Triturate the powders thoroughly together, and add sufficient water to form a plastic mass. Divide this into the required number of pills. Made in this way, a grain pill is not inconveniently large.

The use of simple water as an excipient is, I am told, common in domestic practice in the Southern States. The mass produced in this way possesses too little adhesiveness to render it satisfactory. Tannic acid has been used of late, with a view of diminishing the intense bitterness of the quinine, but has not found favor generally, as far as my observation has extended. How far the known insolubility of the tannate of quinia in water should operate against this combination, is a question for the therapeutist. Conserve of roses, in addition to its bulk, may be objectionable in this as in some other cases, on the score of containing tannic acid, which it does when made from rosa gallica.

The following formula I have used for several years with great satisfaction to myself and to those physicians who have prescribed it. It was first suggested by a southern *medical student.*

Take of sulphate of quinia, 20 grains.
Aromatic sulphuric acid, 15 grains.

Drop the acid into the sulphate of quinia on a tile or slab, and triturate with a spatula until it assumes a pilular consistency; then divide it into the required number of pills. Made in this way, a five grain pill is not inconveniently large.

Although the ingredients when mixed form a fluid, they soon thicken into a paste, and finally become quite solid, and so adhesive as to be readily divided and rolled into pills; care must be taken not to allow the mass to become too dry and brittle before dividing it, as it is liable to do if allowed to remain too long.

In this form a portion of the dissulphate being converted into the soluble neutral sulphate, the preparation more nearly resembles the solution in composition, and is believed to be more rapid and certain in its action.

When it is desired to incorporate other substances in powder with the quiuine thus prepared, they should be added to the mass when it is just so soft that, upon their addition, it will immediately form the proper consistence.

It is not, however, advisable to employ this process when any considerable quantity of other ingredients are prescribed with the quinine, unless a little syrup or honey is also added, to prevent the too rapid hardening and consequent crumbling of the mass.—*American Journal of Pharmrcy.*

